# Determinants of weaning success in patients with prolonged mechanical ventilation

**DOI:** 10.1186/cc7927

**Published:** 2009-06-23

**Authors:** Annalisa Carlucci, Piero Ceriana, Georgios Prinianakis, Francesco Fanfulla, Roberto Colombo, Stefano Nava

**Affiliations:** 1 Respiratory Intensive Care Unit, Fondazione S. Maugeri, IRCCS, Via Maugeri 10, Pavia, 27100, Italy; 2 Department of Intensive Care Medicine, University Hospital of Heraklion, Stavrakia, Heraklion, 71110, Crete, Greece; 3 Service of Clinical Engineering, Fondazione S. Maugeri, IRCCS, Via Maugeri 10, Pavia, 27100, Italy

## Abstract

**Introduction:**

Physiological determinants of weaning success and failure are usually studied in ventilator-supported patients, comparing those who failed a trial of spontaneous breathing with those who tolerated such a trial and were successfully extubated. A major limitation of these studies was that the two groups may be not comparable concerning the severity of the underlying disease and the presence of comorbidities. In this physiological study, we assessed the determinants of weaning success in patients acting as their own control, once they are eventually liberated from the ventilator.

**Methods:**

In 30 stable tracheotomised ventilator-dependent patients admitted to a weaning center inside a respiratory intensive care unit, we recorded the breathing pattern, respiratory mechanics, inspiratory muscle function, and tension-time index of diaphragm (TTdi = Pdisw/Pdi_max _[that is, tidal transdiaphragmatic pressure over maximum transdiaphragmatic pressure] × Ti/Ttot [that is, the inspiratory time over the total breath duration]) at the time of weaning failure (T_0_). The measurements were repeated in all the patients (T_1_) either during a successful weaning trial (successful weaning [SW] group, n = 16) or 5 weeks later, in the case of repeated weaning failure (failed weaning [FW] group, n = 14).

**Results:**

Compared to T_0_, in the FW group at T_1_, significant differences were observed only for a reduction in spontaneous breathing frequency and in TTdi (0.21 ± 0.122 versus 0.14 ± 0.054, *P *= 0.008). SW patients showed a significant increase in Pdi_max _(34.9 ± 18.9 cm H_2_O versus 43.0 ± 20.0, *P *= 0.02) and decrease in Pdisw/Pdi_max _(36.0% ± 15.8% versus 23.1% ± 7.9%, *P *= 0.004).

**Conclusions:**

The recovery of an inadequate inspiratory muscle force could be the major determinant of 'late' weaning success, since this allows the patients to breathe far below the diaphragm fatigue threshold.

## Introduction

In a multicenter study [1], it was found that approximately 15% of patients failed an initial attempt of weaning from mechanical ventilation. This subset of patients usually requires prolonged mechanical ventilation and, for this reason, accounts for about 40% of total intensive care unit (ICU) costs [2]. Repeated weaning failure has been associated with several factors, in particular an imbalance between the increased load and reduced capacity of the inspiratory muscles or cardiovascular impairment or both [3]. Most physiological studies performed to investigate such factors compared patients who at a certain point in time failed a weaning trial with those who did not, so that a potential heterogeneity of the two populations cannot be excluded [4,5]. Two investigations [6,7] were conducted in acutely ill patients who initially could not be weaned from the ventilator but who were later successfully weaned; however, these studies provided only indirect measurement of respiratory muscle function, and the respiratory mechanics was studied during static conditions, while the patients were passively ventilated. In real life, a percentage of ICU patients (approximately 10% to 15%) [8] may fail several weaning attempts before being transferred to a weaning center with the aim of achieving a definitive liberation from the ventilator later on. Up to 50% of these patients may finally be weaned after several weeks [9]. In the present physiological study, we describe the mechanisms of weaning success or failure in difficult-to-wean patients, and for the first time, we use the recordings of respiratory mechanics during a trial of spontaneous breathing in an attempt to understand the underlying mechanism that enables a particular patient to be successfully weaned some time after having failed a previous weaning attempt.

## Materials and methods

Over the course of an 18-month period, 74 consecutive ventilator-dependent patients were admitted to the weaning center of our institution from other hospitals after having failed more than one weaning attempt. Forty-four of these patients were successfully weaned at the first weaning trial, so they were not included in this study. The remaining 30 were included in the investigative protocol that was approved by the institutional ethics committee. Written informed consent was obtained from the patients. All patients were mechanically ventilated through a tracheotomy tube in pressure support ventilation. So that confounding factors could be avoided, patients with primary neuromuscular disorders (that is, Guillain-Barré syndrome, myasthenia gravis, or motor neuron disease) or severe primary cardiomyopathy were excluded *a priori *from the study. We have, however, included those patients with documented ICU-acquired myopathy or polyneuropathy (two for each group), assessed with electrophysiological studies, since they are likely to recover muscle strength over time. Only one patient received glucocorticosteroid treatment during the weaning phase (15 mg of methylprednisolone for 12 days), and none received neuromuscular-blocking agents.

### Experimental procedure

Patients underwent a T-piece trial 48 hours after admission when their clinical conditions were considered stable and the following conditions were met: no fever, pain, or anxiety or hemodynamic compensation and no evident signs of respiratory distress. Patients were disconnected from the ventilator and breathed spontaneously through a T-tube circuit for 1 hour while receiving supplemental oxygen to maintain a peripheral oxygen saturation (SpO_2_) of, on average, 93% to 94%. If this trial was successful, the patients were disconnected from the ventilator. Weaning failure was defined as the occurrence of one of the following at the end of the T-piece trial or within the next 72 hours: (a) oxygen saturation of 90% or less at an inhaled fraction of oxygen (FiO_2_) of 0.5, (b) diaphoresis, (c) evidence of increasing respiratory distress, (d) tachycardia, (e) arrhythmias, (f) hypotension, or (g) increase in arterial partial pressure of carbon dioxide (PaCO_2_) of greater than 20 mm Hg or a pH of less than 7.32 or both. Only patients who failed the weaning trial were recruited in the study. The baseline measurements (T_0_) were performed within 24 hours after the failed weaning attempt once respiratory stability had been achieved by the re-institution of mechanical ventilation.

All of the patients underwent a supervised and standardized rehabilitation program that included proper positioning, passive and active mobilization (that is, leg and arm exercises in bed or in a chair if possible), management of secretion, and (if feasible) ambulation using a walker with the aid of the ventilator and the assistance of a respiratory therapist. Indeed, physiological support or counseling or both was provided. The respiratory therapist was also in charge of the daily screening for a trial of spontaneous breathing according to our internal protocol, which was modified from Ely and colleagues [10]. The limit of 5 weeks to consider a particular patient unweanable was decided based on recent evidence-based guidelines [11]. The authors of those guidelines, in fact, cautioned that patients receiving 'mechanical ventilatory support should not be considered permanently ventilator-dependent until 3 months of weaning attempts have failed'. As a matter of fact, our historical analysis of medical records demonstrated an average of 7 to 8 weeks of ICU stay before admission to our unit. Therefore, we chose the limit of 5 weeks to reach the total 12 weeks for the definition of unweanability [11]. Actually, the second set of measurements (T_1_) was made either 72 hours after the patient had successfully passed a weaning trial (SW group, n = 16, weaned after 10.3 ± 4.4 days) or, in those patients who repeatedly failed the weaning trail (FW group, n = 14), at the end of the fifth week in hospital.

### Physiological measurements

All patients were studied in the semi-recumbent position. During the recording phase, patients breathed an oxygen mixture sufficient to maintain an SpO_2 _value of, on average, 93% to 94%. The following variables were measured: (a) flow (V), measured by a heated pneumotachograph and a differential pressure transducer (Honeywell, Freeport, IL, USA; ± 300 cm H_2_O) connected to the proximal tip of the tracheal cannula; (b) tidal volume (VT) obtained by integration of the flow; (c) inspiratory time (TI), expiratory time (TE), total respiratory time (Ttot), and spontaneous breathing frequency (f) measured from the flow signal; (d) airway pressure (Paw) (Honeywell ± 300 cm H_2_O) measured via a side port between the pneumotachograph and the tracheal cannula; and (e) esophageal (Pes) and gastric (Pga) pressures measured with a balloon catheter system [12]. The proper position of the esophageal balloon was verified using the occlusion test [12]. Transpulmonary (P_L_) and transdiaphragmatic (Pdisw) pressure swings were obtained by subtracting Pes from Paw and Pga, respectively. The dynamic intrinsic positive end-expiratory pressure (PEEPi,dyn) was estimated as described by Appendini and colleagues [13].

The magnitude of the inspiratory muscle effort was estimated from the pressure time product for the diaphragm (PTPdi) and for the inspiratory muscles *in toto *(esophageal pressure time product, or PTPes). The pressure time integrals were calculated per breath and per minute [14]. Dynamic lung compliance (C_Ldyn_) and pulmonary resistance at midinspiratory volume (R_L_) were computed from P_L_, V, and VT records as previously described [13].

Physiological signals were collected for 5 minutes at the end of the spontaneous breathing trial. At the tip of the tracheotomy tube, we inserted a device consisting of a rigid T-tube with a unidirectional valve set on the expiratory line in order to measure the maximum inspiratory pressure (MIP) and maximum trandiaphragmatic pressure (Pdi_max_). Measurements were performed according to the method previously described [13]. The tension-time index of the diaphragm (TTdi) was computed using Pdi_max _according to the method of Bellemare and Grassino [15,16]: TTdi = Pdisw/Pdi_max _× Ti/Ttot. Mean inspiratory Pdisw was also expressed as a fraction of Pdi_max_.

### Data analysis

Results are presented as the mean and standard deviation. The Kolmogorov-Smirnov statistic with a Lilliefors significance level and Shapiro-Wilk tests were used to test the normality of distribution of all of the considered variables. Differences in anthropometric or physiological data between the two groups of patients were assessed by one-way analysis of variance (ANOVA), whereas differences in categorized variables were assessed by chi-square test. Two-way ANOVA analysis for repeated measures was performed to analyze changes in pulmonary function parameters over time between the two groups of patients considered. The Tukey honestly significant differences test and the Scheffé test were used to compare differences between groups and within groups, respectively. We performed a multifactorial ANOVA analysis for repeated measures to analyze changes in the muscle function indices according to the type of disease and the outcome of weaning procedures. A *P *value of less than 0.05 was considered statistically significant. All of the analyses were performed using the STATISTICA/W statistical package (StatSoft, Inc., Tulsa, Oklahoma, USA).

## Results

Patients' characteristics are shown in Table 1. No significant differences were found in the variables considered. The distribution of causes responsible for onset of mechanical ventilation was not different in the two groups. All of the variables considered in the analysis were normally distributed according to the Kolmogorov-Smirnov test. All of the patients underwent the two sets of measurements of respiratory mechanics (that is, at T_0 _and either at the time of weaning or after 5 weeks, T_1_). Liberation from mechanical ventilation occurred in the SW group after 11.4 ± 6.3 days. Table 2 illustrates the data of respiratory mechanics and ventilatory pattern at enrollment in the two groups of patients. The FW and SW groups were similar for all respiratory variables except for the respiratory rate, Pdisw/Pdi_max_, and TTdi, which were significantly higher in the FW group. Table 3 shows a comparison between the two groups for the variables recorded at the end of the study. Compared with the FW group, the SW group maintained a significantly lower Pdisw/Pdi_max _ratio and TTdi but also showed an improved Pdi_max _and MIP, together with a reduced f/VT ratio.

**Table 1:** Patients' characteristics at enrollment

	Successful weaning group (n = 16)	Failed weaning group (n = 14)	*P *value
Gender, male/female	9/7	10/4	NS
Age, years	67.6 ± 13.5	70.9 ± 11	NS
Body mass index	24 ± 5.6	21.6 ± 2.6	NS
SAPS II	29.6 ± 7.3	31.6 ± 6	NS
Diagnosis			NS
Post-cardiac surgery	5	4	
ALI/ARDS	5	2	
COPD exacerbation	6	8	
Duration of MV at the time of the study^a^	37.5 ± 19.6 (25–40)	48.9 ± 26.9 (30–60)	NS

^a^The 25th to 75th percentiles are reported in parentheses. ALI/ARDS, acute lung injury/acute respiratory distress syndrome; COPD, chronic obstructive pulmonary disease; MV, mechanical ventilation; NS, not significant; SAPS II, Simplified Acute Physiology Score II.

**Table 2:** Ventilatory pattern and respiratory mechanics at enrollment

	Successful weaning group (n = 16)	Failed weaning group (n = 14)	*P *value
Ventilatory pattern			
VT, mL	336.5 ± 158.3	299.5 ± 213.4	NS
f, breaths/min	26.1 ± 7.5	32.4 ± 5.2	0.01
f/VT	109.4 ± 74.5	173.9 ± 103.4	NS
Respiratory mechanics			
C_Ldyn_, L/cm H_2_O	0.049 ± 0.032	0.051 ± 0.035	NS
R_L_, cm H_2_O/L per s	13.4 ± 9.0	12.9 ± 9.4	NS
PEEPi,dyn, cm H_2_O	1.93 ± 1.36	2.7 ± 3.1	NS
Inspiratory muscle function			
MIP, cm H_2_O	45.2 ± 19.5	32.7 ± 18.2	NS
Pdi_max_, cm H_2_O	34.9 ± 18.9	25.4 ± 17.3	NS
Pdisw/Pdi_max_, percentage	36.1 ± 15.8	54.4 ± 25.5	0.02
PTPdi/min, cm H_2_O/s	235.8 ± 126.9	268.0 ± 234.8	NS
TTdi	0.13 ± 0.065	0.21 ± 0.12	0.02

C_Ldyn_, dynamic lung compliance; f, spontaneous breathing frequency; MIP, maximum inspiratory pressure; NS, not significant; Pdi_max_, maximum transdiaphragmatic pressure; Pdisw, tidal diaphragmatic pressure; PEEPi,dyn, dynamic intrinsic positive end-expiratory pressure; PTPdi/min, pressure time product of the diaphragm per minute; R_L_, pulmonary resistance; TTdi, tension-time index of diaphragm; VT, tidal volume.

**Table 3:** Ventilatory pattern and respiratory mechanics at the end of the study

	Successful weaning group (n = 16)	Failed weaning group (n = 14)	*P *value
Ventilatory pattern			
VT, mL	385.8 ± 132.2	289.3 ± 138.4	NS
f, breaths/min	22.6 ± 6.0	27.4 ± 7.3	NS
f/VT	74.1 ± 44.0	148.2 ± 121.4	0.03
Respiratory mechanics			
C_Ldyn_, L/cm H_2_O	0.067 ± 0.033	0.049 ± 0.024	NS
R_L_, cm H_2_O/L per s	8.8 ± 5.8	14.4 ± 14.2	NS
PEEPi,dyn, cm H_2_O	1.5 ± 1.0	1.7 ± 1.66	NS
Inspiratory muscle function			
MIP, cm H_2_O	57.3 ± 18.2	38.6 ± 13.5	0.001
Pdi_max_, cm H_2_O	43.0 ± 20.0	27.7 ± 12.5	0.01
Pdi_sw_/Pdi_max_, percentage	23.1 ± 7.9	42.5 ± 22.9	0.003
PTPdi/min, cm H_2_O/s	194.1 ± 84.8	216.2 ± 136.8	NS
TTdi	0.08 ± 0.029	0.14 ± 0.054	0.009

C_Ldyn_, dynamic lung compliance; f, spontaneous breathing frequency; MIP, maximum inspiratory pressure; NS, not significant; Pdi_max_, maximum transdiaphragmatic pressure; Pdisw, tidal diaphragmatic pressure; PEEPi,dyn, dynamic intrinsic positive end-expiratory pressure; PTPdi/min, pressure time product of the diaphragm per minute; R_L_, pulmonary resistance; TTdi, tension-time index of diaphragm; VT, tidal volume.

**Figure 1:**
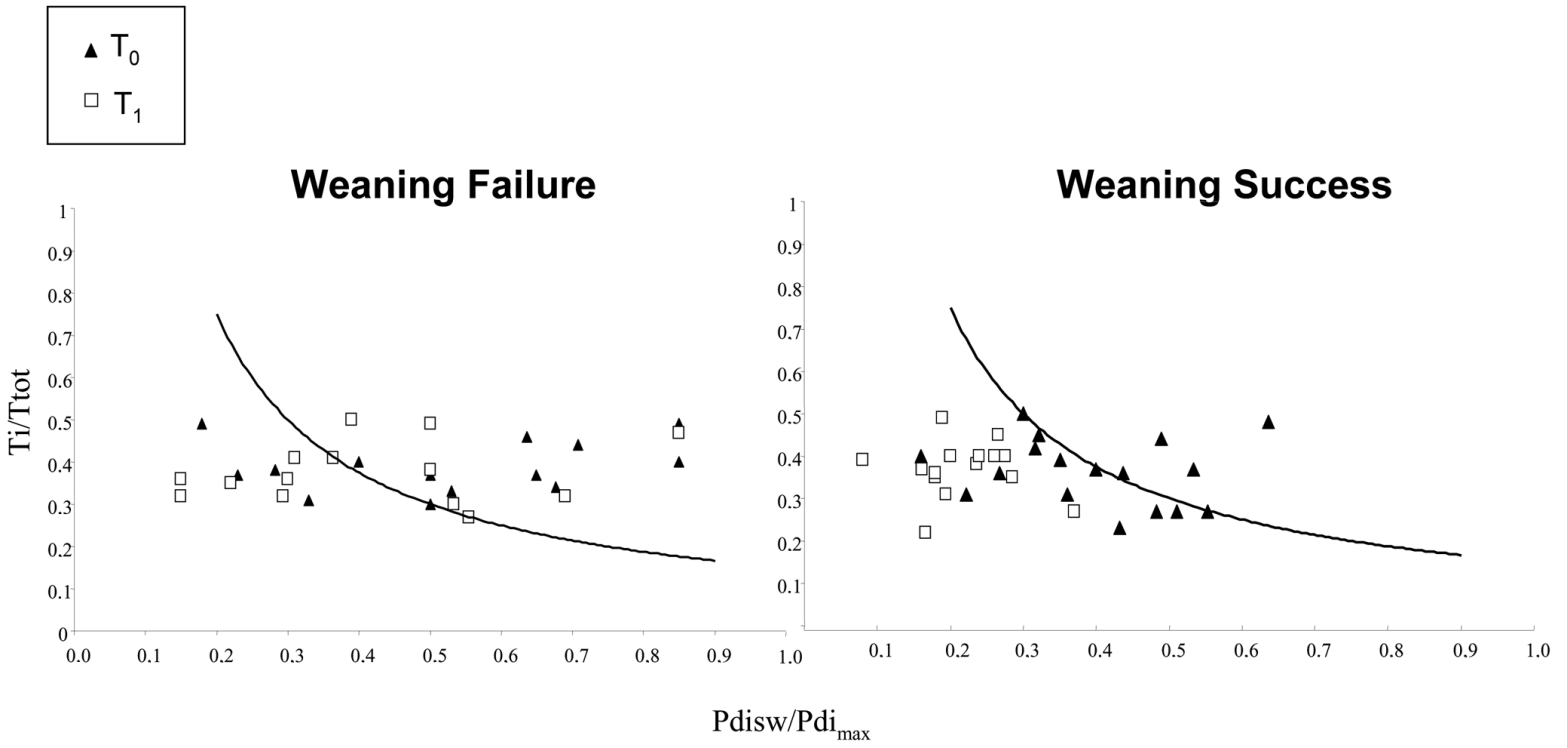
Tension-time diaphragmatic index at T_0 _(black triangles) and T_1 _(white squares) in the weaned and unweaned groups. Pdisw/Pdi_max_, ratio of tidal diaphragmatic pressure to maximum transdiaphagmatic pressure. Ti/Ttot, inspiratory time expressed as a fraction of the total respiratory cycle duration.

As shown in Table 4, the two-way ANOVA analysis for repeated measures found statistically significant differences between the two groups of patients for MIP (*P *= 0.04), Pdisw/Pdi_max _(*P *= 0.004), and TTdi (*P *= 0.03). Significant differences within groups were found for Pdi_max _(*P *= 0.02) and Pdisw/Pdi_max _(*P *= 0.004) in the SW group and for TTdi in the FW group (*P *= 0.008). TTdi changes over time in weaning success and failure patients are shown in Figure 1. A multifactorial ANOVA analysis for repeated measures was performed to analyze changes in the muscle function indices according to the type of disease and the outcome of weaning procedures. The type of disease has an independent role only for the changes in Pdi_max _that we observed between T_0 _and T_1 _in SW patients (ANOVA F 6.7, *P *= 0.005), as shown in Table 5. Four patients died after the end of the study, during the hospital stay. A statistically significant association was found between mortality and weaning outcome since all of the patients who died were in the FW group (chi-square 5.27, *P *= 0.02).

**Table 4:** Inspiratory muscle function and effort in weaned and unweaned patients

Group	MIP, cm H_2_O	Pdi_max_, cm H_2_O	Pdisw/Pdi_max_, percentage	TTdi
Successful weaning				
T_0_	45.2 ± 19.5	34.9 ± 18.9^a^	36.0 ± 15.8^a^	0.13 ± 0.065^b^
T_1_	57.3 ± 18.2^b^	43.0 ± 20.04^a^	23.1 ± 7.9^a,b^	0.08 ± 0.029
Failed weaning				
T_0_	32.7 ± 18.2	25.4 ± 17.3	54.4 ± 25.5	0.21 ± 0.122^a,b^
T_1_	38.6 ± 13.5^b^	27.7 ± 12.5	42.5 ± 22.9^b^	0.14 ± 0.054^a^

^a^*P *< 0.05 differences for each variable within groups; ^b^*P *< 0.05 differences for each variable between groups. MIP, maximum inspiratory pressure; Pdi_max_, maximum transdiaphragmatic pressure; Pdisw, tidal diaphragmatic pressure; TTdi, tension-time index of diaphragm.

**Table 5:** Changes in Pdi_max _(cm H_2_O) over time in successful weaning and failed weaning patients according to the baseline disease

Group	T_0_	T_1_
Successful weaning		
COPD	50.2 ± 11.9 (4.9)	59.3 ± 13.5 (5.5)^a^
Non-COPD	28.83 ± 16.5 (5.2)	33.2 ± 16.9
Failed weaning		
COPD	32.1 ± 17.6 (6.2)	32.1 ± 10.6 (3.7)^a^
Non-COPD	16.5 ± 13.5^a ^(5.5)	22 ± 13.5 (5.5)^a^

Data are presented as the mean ± standard deviation (standard error). COPD, chronic obstructive pulmonary disease; Pdi_max_, maximum transdiaphragmatic pressure.

^a^, post doc analysis *P *< 0.05.

## Discussion

This study shows that ventilator-dependent patients finally achieved definitive liberation from mechanical ventilation through a physiological mechanism that led to a significant increase in the force-generating capacity of the diaphragm (Pdi_max_). This allowed an improvement in the load/capacity balance (Pdisw/Pdi_max_) and consequently a reduction of the TTdi. As a matter of fact, the TTdi returned to well below the so-called fatigue threshold (0.15 to 0.18) in the SW group, whereas it was near the fatigue threshold in the FW group.

Although the mechanisms of weaning success or failure have been studied quite extensively, this is the first physiological investigation that used the patients as their own control in a before-and-after fashion and, more importantly, that employed the recording of respiratory mechanics during a trial of spontaneous breathing. This is particularly important since the passive measurements of respiratory mechanisms obtained in previous studies are only surrogates of the real-life situation in which a patient is asked to breathe totally without support.

The mechanisms underlying the inability to sustain spontaneous ventilation in ventilator-dependent patients have been only partially investigated. Jubran and Tobin [4] first reported systematic measurements of respiratory muscle function and respiratory mechanics in patients with chronic obstructive pulmonary disease (COPD) who failed a trial of spontaneous breathing, and compared the results with those obtained in COPD patients successfully extubated at the first attempt. These authors showed that the major determinant between a successful and an unsuccessful weaning trial was a change in breathing pattern rather than an intrinsic abnormality in pulmonary mechanics. Later, Purro and colleagues [5] studied the physiological determinants of ventilator dependency in stable COPD and post-cardiac surgery patients who failed repeated weaning attempts, comparing these patients with spontaneously breathing, but previously ventilated, patients matched for age and disease. The authors found that ventilator-dependent patients showed a higher load/capacity balance and a greater effective inspiratory impedance than a group of tracheotomized patients liberated from mechanical ventilation more than 15 months before. Unfortunately, the lack of measurements of respiratory mechanics and inspiratory muscle function at the time of the definitive independence from mechanical ventilation makes the comparison of the cases (ventilator-dependent patients) with controls difficult to interpret. Two successive studies used a protocol similar to that used in our study, but in critically ill patients admitted to the ICU [6,7]. Vassilakopoulos and colleagues [7] studied one group of patients who initially had failed to wean from mechanical ventilation but had successful weaning on a later occasion. Patients were studied while most of them were still ventilated through an endotracheal tube and a clinical stability had been required for only the preceding 12 hours. In that study, respiratory muscle function was measured non-invasively, while respiratory mechanics was studied in static condition with patients ventilated with control mechanical ventilation and constant inspiratory flow. In the same year, Capdevila and colleagues [6] published a study in which 17 difficult-to-wean patients in the ICU were eventually divided into those successfully (11 patients) and unsuccessfully (6 patients) weaned. However, no direct measurements of respiratory mechanics and respiratory muscle function were performed since they relied of non-invasive methods, mainly derived from the occlusion pressure (P_0.1_) signal.

The work of Vassilakopoulos and colleagues [7] included patients who initially failed a weaning trial and followed them to the point of successful weaning. Compared with SW patients, FW patients had greater total resistance, intrinsic PEEP, dynamic hyperinflation, ratio of mean to maximum inspiratory pressure, and tension-time index (TTI) and less MIP and a breathing pattern that was more rapid and shallow. In a regression analysis, these authors found that TTI and f/VT were the only significant variables that predicted weaning success. Capdevila and colleagues [6] conducted a similar study but looked at physiological variables at 24-hour intervals to describe the temporal evolution of difficult-to-wean patients. In this latter study, the authors did not record data at T_0 _and so they described physiological outcomes based on whether patients were successfully weaned or not. They found that weaning failure was associated with longer periods of ventilation before weaning, high breathing frequency and tracheal P_0.1_, minute ventilation, and persistently high PaCO_2 _and intrinsic PEEP. They also found that the TTI remained in the fatigue zone. Conversely, SW patients normalized their breathing pattern and were able to reduce their tracheal P_0.1 _and TTI.

Our study provides, for the first time, a direct measurement of respiratory muscle function in the same group of patients, so that they may be considered their own control, minimizing other confounding variables that may be present when comparing two different groups of patients (that is, weaning failure or success). The recording of active respiratory mechanics is also different from passive recordings since the latter represent a surrogate of the 'real life' picture once the patients are disconnected from the ventilator. The values of respiratory mechanics (that is, compliance and resistance) have also been shown to vary consistently when recorded with the two methods. For example, during the 'passive' recordings, the values are likely to be influenced by the ventilator settings (that is, set breathing frequency). Indeed, the present investigation was performed on a subset of patients far from an acute episode and therefore considered 'true' ventilator-dependent patients. Although this subset of patients may account for 10% to 15% of the whole ICU population, little attention has been paid to the mechanisms eventually leading to liberation from the ventilatory support, even after weeks of mechanical ventilation.

In our study, apart from a small but significant reduction in respiratory rate in the FW group, no differences were observed in the breathing pattern between T_0 _and T_1_, suggesting that the ventilatory strategy adopted by the patients during a T-piece trial is not the main determinant of weaning success, as described in more acutely ill patients [4]. No major changes were observed in the SW group between T_0 _and T_1 _in the parameters of diaphragmatic effort, such as tidal Pdisw and PTPdi. The main determinant of weaning success was therefore related to the significant improvement of diaphragmatic inotropism at the time of gaining independence from mechanical ventilation.

Several factors may be responsible for the reduced Pdi_max _observed in ventilator-dependent patients. Age, hypercapnia, hypoxia, malnutrition, treatment with corticosteroids or other agents, cardiovascular problems, and inactivity may all lead to an impairment of diaphragmatic performance [17-19]. Most importantly, there is compelling evidence that mechanical ventilation *per se*, especially if it is protracted and delivered in a controlled mode, may lead to a decreased force-generating capacity of the diaphragm, associated with muscle atrophy, oxidative stress, and also wasting and damage [20-23]. A reduction in Pdi_max _has also been specifically addressed by Laghi and colleagues [24] in patients at the beginning of a failed weaning attempt. Indeed, impaired diaphragmatic function may be a major cause of weaning failure, as assessed using cervical magnetic stimulation, in a population of post-surgical patients [25]. The improvement in Pdi_max _at T_1 _in our SW patients may be related to several factors. The comprehensive rehabilitation program that the patients underwent can be associated with a significant improvement in skeletal force and diaphragm pressure, as reported by two studies performed in ICU patients [26,27]. Finally, an uncontrolled study demonstrated that the use of selective inspiratory muscle training may facilitate weaning in ventilator-dependent patients [28]. It is more difficult to explain why Pdi_max _did not improve in about half of our patients, resulting in failed weaning, but it might be that the diaphragm fibers were irreversibly damaged by the more prolonged ventilation (Table 1).

The large majority of patients can be liberated from the ventilator after the first weaning attempt. In those patients with weaning difficulties, it has been suggested that the f/VT ratio, which may give an estimate of the capability of sustaining a spontaneous breathing trial, be monitored daily. We have also found a statistically significant difference in the f/VT ratio between the weaned and unweaned group, which is in keeping with the literature. Therefore, one may claim that the rapid shallow breathing index may be a surrogate of the most complex-to-measure Pdi/Pdi_max _or TTdi. There are, however, two important comments to made. At enrollment in the study, both Pdi/Pdi_max _and TTdi were different between the groups, reflecting the fact that these parameters are 'quantitatively' more accurate in discriminating the 'potential reserve' of a patient, even at the time of a weaning failure. Indeed, the f/VT ratio is probably more influenced by psychological reasons and, last but not least, may be misleading in those patients who usually do not increase dramatically the breathing frequency, to avoid the phenomenon of dynamic hyperinflation that is a consequence of an elevated breathing frequency [29]. For these reasons, we suggest that, whenever feasible and possible, the measurements of active respiratory mechanics be recorded to give the clinician better insight into the weaning possibilities of a certain ventilator-dependent patient.

## Conclusions

Using invasive and direct measurements of 'active' respiratory mechanics and diaphragmatic function, we have shown that stable ventilator-dependent patients who have initially failed more than one weaning attempt are characterized by a high load/capacity balance, especially due to a reduced Pdi_max _rather than to an excessive workload, so that once they are breathing spontaneously, they are placed above the threshold of diaphragm fatigue. The re-institution of a higher Pdi_max _was associated with definitive weaning from the ventilator and with a downward shift in the fatigue threshold. Conversely, the inotropic characteristic of the diaphragm did not improve in patients who could not be weaned.

## Key messages

• In this study, we compared the physiological changes that enable a particular patient to be successfully weaned some time after having failed a previous weaning attempt.

• Patients who were successfully weaned afterward showed an improved maximum transdiaphragmatic pressure and a better load/capacity ratio compared with the first unsuccessful weaning attempt.

• At the time of the first unsuccessful weaning attempt, both groups, weaned and unweaned patients, showed a tension-time index of the diaphragm (TTdi) that was above the so-called fatigue threshold.

• Both groups, weaned and unweaned patients, showed a reduced TTdi with time, but this remained above the fatigue threshold in the unweaned group.

• The recovery of an inadequate inspiratory muscle force could be the major determinant of 'late' weaning success since this allows the patients to breathe far below the diaphragm fatigue threshold.

## Abbreviations

ANOVA: analysis of variance; COPD: chronic obstructive pulmonary disease; f: spontaneous breathing frequency; FW: failed weaning; ICU: intensive care unit; MIP: maximum inspiratory pressure; P_0.1_: occlusion pressure; PaCO_2_: arterial partial pressure of carbon dioxide; Paw: airway pressure; Pdi_max_: maximum transdiaphragmatic pressure; Pdisw: tidal diaphragmatic pressure; PEEP: positive end-expiratory pressure; Pes: esophageal pressure; Pga: gastric pressure; P_L_: transpulmonary pressure; PTPdi: diaphragmatic pressure time product; SpO_2_: peripheral oxygen saturation; SW: successful weaning; Ti: inspiratory time; TTdi: tension-time index of the diaphragm; TTI: tension-time index; Ttot: total breath duration; V: flow; VT: tidal volume.

## Competing interests

The authors declare that they have no competing interests.

## Authors' contributions

AC conceived of the study and participated in the collection of data and the drafting of the manuscript. PC participated in the study design and the collection and interpretation of data. GP participated in the study design and the collection of data. FF participated in the study design and the statistical analysis. RC participated in the acquisition and analysis of data. SN participated in the study design, coordinated the collection of data, participated in data analysis and interpretation, and helped to draft the manuscript. All authors read and approved the final manuscript.

## References

[B1] Esteban A, Anzueto A, Frutos F, Alia I, Brochard L, Stewart TE, Benito S, Epstein SK, Apezteguia C, Nightingale P, Arroliga AC, Tobin MJ, Mechanical Ventilation International Study Group (2002). Characteristics and outcomes in adult patients receiving mechanical ventilation: a 28-day international study. JAMA.

[B2] Wagner DP (1989). Economics of prolonged mechanical ventilation. Am Rev Respir Dis.

[B3] Epstein SK (2002). Decision to extubate. Intensive Care Med.

[B4] Jubran A, Tobin MJ (1997). Pathophysiologic basis of acute respiratory distress in patients who fail a trial of weaning from mechanical ventilation. Am J Respir Crit Care Med.

[B5] Purro A, Appendini L, De Gaetano A, Gudjonsdottir M, Donner CF, Rossi A (2000). Physiologic determinant of ventilator dependence in long-term mechanically ventilated patients. Am J Respir Crit Care Med.

[B6] Capdevila X, Perrigault PF, Ramonatxo M, Roustan JP, Peray P, d'Athis F, Prefaut C (1998). Changes in breathing pattern and respiratory muscle performance parameters during difficult weaning. Crit Care Med.

[B7] Vassilakopoulos T, Zakynthinos S, Roussos C (1998). The tension-time index and the frequency/tidal volume ratio are the major pathophysiologic determinants of weaning failure and success. Am J Respir Crit Care Med.

[B8] Boles JM, Bion J, Connors A, Herridge M, Marsh B, Melot C, Pearl R, Silverman H, Stanchina M, Vieillard-Baron A, Welte T (2007). Weaning from mechanical ventilation. Eur Respir J.

[B9] Scheinhorn DJ, Hassenpflug MS, Votto JJ, Chao DC, Epstein SK, Doig GS, Knight EB, Petrak RA, Ventilation Outcomes Study Group (2007). Post-ICU mechanical ventilation at 23 long-term care hospitals: a multicenter outcomes study. Chest.

[B10] Ely EW, Baker AM, Dunagan DP, Burke HL, Smith AC, Kelly PT, Johnson MM, Browder RW, Bowton DL, Haponik EH (1996). Effect on the duration of mechanical ventilation of identifying patients capable of breathing spontaneously. N Engl J Med.

[B11] MacIntyre NR, Cook DJ, Ely EW, Epstein SK, Fink JB, Heffner JE, Hess D, Hubmayer RD, Scheinhorn DJ, American College of Chest Physicians; American Association for Respiratory Care; American College of Critical Care Medicine (2001). Evidence-based guidelines for weaning and discontinuing ventilatory support: a collective task force facilitated by the American College of Chest Physicians; the American Association for Respiratory Care; and the American College of Critical Care Medicine. Chest.

[B12] Baydur A, Behrakis PK, Zin WA, Jaeger M, Milic-Emili J (1982). A simple method for assessing the validity of the oesophageal balloon technique. Am Rev Respir Dis.

[B13] Appendini L, Purro A, Patessio A, Zanaboni S, Carone M, Spada E, Donner CF, Rossi A (1996). Partitioning of inspiratory muscle work load and pressure assistance in ventilator-dependent patients with COPD. Am J Respir Crit Care Med.

[B14] Sassoon CS, Light RW, Lodia R, Sieck GC, Mahutte CK (1991). Pressure-time product during continuous positive airway pressure, pressure support ventilation, and T-piece during weaning from mechanical ventilation. Am Rev Respir Dis.

[B15] Bellemare F, Grassino A (1982). Effect of pressure and timing of contraction on human diaphragm fatigue. J Appl Physiol.

[B16] Bellemare F, Grassino A (1983). Force reserve of the diaphragm in patients with chronicobstructive pulmonary disease. J Appl Physiol.

[B17] Juan G, Calverley P, Talamo C, Schnader J, Roussos C (1984). Effect of carbon dioxide ondiaphragmatic function in human beings. N Engl J Med.

[B18] Lewis MI, Sieck GC (1992). Effect of acute nutritional deprivation on diaphragmatic structure and function in adolescent rats. J Appl Physiol.

[B19] Nava S, Gayan-Ramirez G, Rollier H, Bisschop A, Dom R, de Bock V, Decramer M (1996). Effects of acute steroid administration on ventilatory and peripheral muscles in rats. Am J Respir Crit Care Med.

[B20] Le Bourdelles G, Viires N, Boczkowski J, Seta N, Pavlovic D, Aubier M (1994). Effects of mechanical ventilation on diaphragmatic contractile properties in rats. Am J Respir Crit Care Med.

[B21] Hund EF, Fogel W, Krieger D, DeGeorgia M, Hacke W (1996). Critical illness polyneuropathy: clinical findings and outcomes of a frequent cause of neuromuscular weaning failure. Crit Care Med.

[B22] Jaber S, Sebbane M, Koechlin C, Hayot M, Capdevila X, Eledjam JJ, Prefaut C, Ramonatxo M, Matecki S (2005). Effects of short vs. prolonged mechanical ventilation on antioxidant systems in piglet diaphragm. Intensive Care Med.

[B23] Levine S, Nguyen T, Taylor N, Friscia ME, Budak MT, Rothenberg P, Zhu J, Sachdeva R, Sonnad S, Kaiser LR, Rubinstein NA, Powers SK, Shrager JB (2008). Rapid disuse atrophy of diaphragm fibers in mechanically ventilated humans. N Engl J Med.

[B24] Laghi F, Cattapan SE, Jubran A, Parthasarathy S, Warshawsky P, Choi YS, Tobin MJ (2003). Is weaning failure caused by low-frequency fatigue of the diaphragm?. Am J Respir Crit Care Med.

[B25] Buscher H, Valta P, Boie T, Hinz J, Moerer O, Sydow M, Mudaliar MY, Burchardi H (2005). Assessment of diaphragmatic function with cervical magnetic stimulation in critically ill patients. Anaesth Intensive Care.

[B26] Martin UJ, Hincapie L, Nimchuk M, Gaughan J, Criner GJ (2005). Impact of whole-body rehabilitation in patients receiving chronic mechanical ventilation. Crit Care Med.

[B27] Nava S (1998). Rehabilitation of patients admitted to a respiratory intensive care unit. Arch Phys Med Rehabil.

[B28] Martin AD, Davenport PD, Franceschi AC, Harman E (2002). Use of inspiratory muscle strength training to facilitate ventilator weaning: a series of 10 consecutive patients. Chest.

[B29] Conti G, Montini L, Pennisi MA, Cavaliere F, Arcangeli A, Bocci MG, Proietti R, Antonelli M (2004). A prospective, blinded evaluation of indexes proposed to predict weaning from mechanical ventilation. Intensive Care Med.

